# Deformation Mechanism and Control of In-Situ Assembling Caisson Technology in Soft Soil Area under Field Measurement and Numerical Simulation

**DOI:** 10.3390/ma16031125

**Published:** 2023-01-28

**Authors:** Jie Zhou, Chengjun Liu, Jie Xu, Zhenguang Zhang, Zeyao Li

**Affiliations:** 1 Department of Geotechnical Engineering, Tongji University, Shanghai 200092, China; 2 Shanghai Road and Bridge Group Co. Ltd., Shanghai 200433, China

**Keywords:** in-situ assembling caisson, VSM construction method, ground settlement, deep layered deformation, field measurement, stability

## Abstract

With urban space becoming much more crowded, the construction of underground spaces continues to expand to deeper, and the requirements for the large depth and minor deformation in urban engineering construction are getting more urgent. A new kind of in-situ assembling caisson technology (called VSM) is a vertical shaft method (VSM), which excavates the stratum under water with a mechanical arm and assembles the prefabricated caisson segments at the same time. This paper takes the Shanghai Zhuyuan Bailonggang Sewage Connecting Pipe Project as an example, which is the first construction project in the soft soil area, such as Shanghai, and makes a technical analysis of the VSM by comparing the field measurement and numerical simulation. Ground settlements and layered deep displacements were monitored in the field measurement during the VSM construction. It shows that the maximum ground settlement caused by the VSM is 15.2 mm and the maximum horizontal displacement is 3.74 mm. The influence range of the shaft excavation on the ground settlement is about 30 m away from the shaft center. The results demonstrate that the VSM construction has great applicability in the soft soil area. A finite element simulation model of the VSM shaft is established and verified by field measurement. There is a certain error between the traditional theoretical calculation by analogy to the common retaining walls of the deep foundation pit and the measured results, while the simulation results are relatively consistent with field measurements. The reasons for the difference are well-analyzed. Finally, the effects of the VSM construction method on the engineering environment are analyzed, and the suggestions for deformation control in the future are put forward.

## 1. Introduction

A shaft is an important structural form of underground space construction. In recent years, with the continuous development of urban construction, underground space excavation has had a complex and diversified development. Underground engineering construction puts forward higher and higher requirements for shaft excavation.

The VSM applies to the excavation of small diameter shafts in urban areas and does not require dewatering. It has the advantages of deep excavation depth, small construction disturbance, fast construction speed, small site use, high economic efficiency, and strong stratum applicability. This kind of method has been used in shaft engineering in Europe, the United States [1], Singapore [2], and other places. The VSM construction method is mainly used in subway ventilation shaft construction and presently has a maximum excavation depth of 115.2 m [3,4].

There are relatively few engineering applications of the VSM in China [5]; Zhang et al. [6], Huang et al. [7], Jiang et al. [8] took the ultra-deep prefabricated shaft project in Jianye District, Nanjing as an example and mainly introduced the key technology of ultra-deep prefabricated shaft undrained construction in water-rich sand stratum; but the requirements for the surrounding engineering environment is not very high since it is away from the downtown. In general, there are relatively few studies of the VSM construction method in soft clay area, such as Shanghai, as well as the impact of this new technology on the engineering environment. 

Even though few specific numerical simulations of in-situ assembling caisson technology are conducted at present, previous research on the excavation of deep foundation and traditional caisson can provide good significance. Ma [9] studied ground settlement and the stability of the retaining wall deformation in the process of excavation by COMSOL and also made a comparative analysis with the monitoring data during the excavation of a deep foundation pit in Shanghai. Lin et al. [10] established a numerical simulation model by FLAC3D to study the pile displacement and ground settlement with the exaction time under different computation boundaries for considering creep and seepage. Shi et al. [11] focused on the mechanical characteristics and cracking control of a large diameter caisson in the initial sinking stage in the FEM numerical simulation by ANSYS. Zhao et al. [12] studied the stress and deformation performance of an anchor caisson foundation in sands by model tests and the numerical simulation in PLAXIS 3D; and the mechanism on the interaction of the soil-structure (anchor caisson wall) was also analyzed. Different from deep foundation construction with underground diaphragm wall, or traditional caisson, the construction speed is much faster and the construction condition is underwater; generally, the construction depth can also be much deeper. Most importantly, nowadays deep shafts are rarely constructed by traditional caisson in urban cities due to the severe engineering environmental impact, instead deep foundation pits by underground diaphragm wall construction are always used. However, the leakage problem greatly impedes the construction depth induced by the diaphragm wall quality; simultaneously, dewatering is really not environmentally-friendly in the soft soil area. Therefore, the deformation mechanism and control of this new in-situ assembling caisson technology should be specifically analyzed for potential broad utilization, especially in Shanghai, where thick, soft mucky clays are widely distributed. 

Taking the first engineering project in Shanghai as an example, the 17# shaft in the Shanghai Zhuyuan Bailonggang Sewage Connecting Pipe Project, where a specifically designed field measurement of the ground settlement and the deep layered deformation of surrounding soils are analyzed in this paper. Simultaneously, the traditional theoretical calculations for design and numerical simulation are both conducted to compare with field measurements. In addition, the effects of the VSM construction method on the engineering environment are analyzed, and the suggestions for the deformation control in the future are put forward.

## 2. VSM Construction Technology

**Figure 1:**
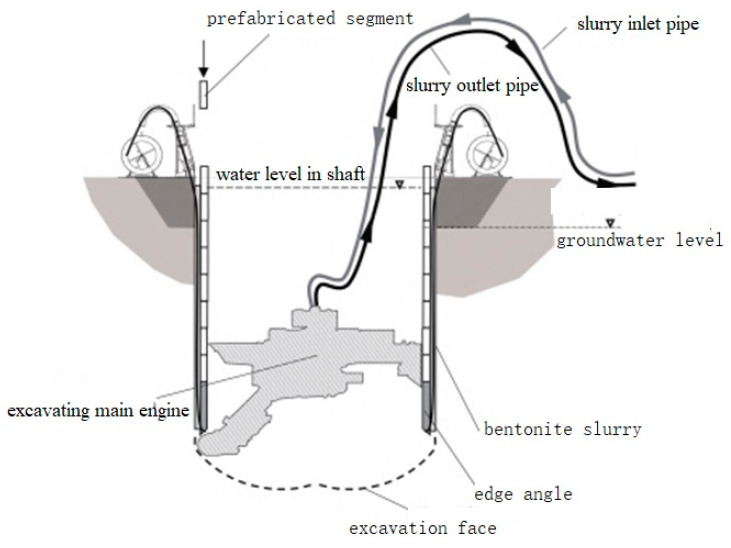
Schematic diagram of VSM construction method.

The VSM construction method is a submerged in-situ assembling shaft excavation construction method in which the mechanical arm is used to excavate the stratum underwater and assemble the prefabricated caisson segments at the same time. The VSM construction equipment (as shown in Figure 1) is mainly composed of the excavating main engine, slurry inlet pipe, slurry outlet pipe, prefabricated caisson segment, and sinking unit.

While the mechanical arm attached to the excavating main engine is excavating the soil in the shaft, prefabricated caisson segments are being assembled at the ground surface, and the sinking unit is being operated to sink the caisson segments. Bentonite is grouted between the prefabricated segments and the soil during the sinking to mostly reduce friction resistance. During the process of excavation and sinking, water should be injected into the shaft in time to ensure that the water level in the shaft is always higher than the groundwater level to keep excavation stability. Each prefabricated caisson segment is connected with each other by radial longitudinal bolts. During the sinking of the prefabricated caisson segments, the sinking unit is always connected to the bottom edge of the first prefabricated caisson segment through steel strands to precisely control the sinking speed. Bentonite grouting holes are designed at the lower part of the shaft, which is connected to the bentonite grouting pipe. The process of excavation, assembling, and sinking is cycled until the shaft excavation reaches the design depth.

Once the excavation reaches the design depth, the bottom sealing shall be operated by pouring concrete underwater to form concrete slab. Then, the cement slurry is used to replace the bentonite filled outside the prefabricated caisson segments. After that, the water in the shaft is pumped, and the inner wall of shaft is flushed by a high-pressure water gun. Finally, shaft floor slab construction works, such as binding steel bars and pouring concrete, are completed.

Compared with other shaft construction methods, the VSM has the following advantages:

(1) The diameter range of the shaft is 4.5~18 m.
(2) The construction depth can reach over 120 m below the groundwater level.
(3) Prefabricated caisson segments greatly increase the stability of the shaft.
(4) VSM can be operated remotely.
(5) Surrounding environmentally-friendly by underwater sinking without lowering the groundwater level.
(6) Fast construction speed and controllable structure sinking process.
(7) The construction site is small.

## 3. Project Overviews

Zhuyuan Bailonggang Sewage Connecting Pipe Project is located in the Pudong New Area, Shanghai. The total length of connecting pipeline is about 19.8 km, which is constructed by the shield method and pipe jacking method.

**Figure 2:**
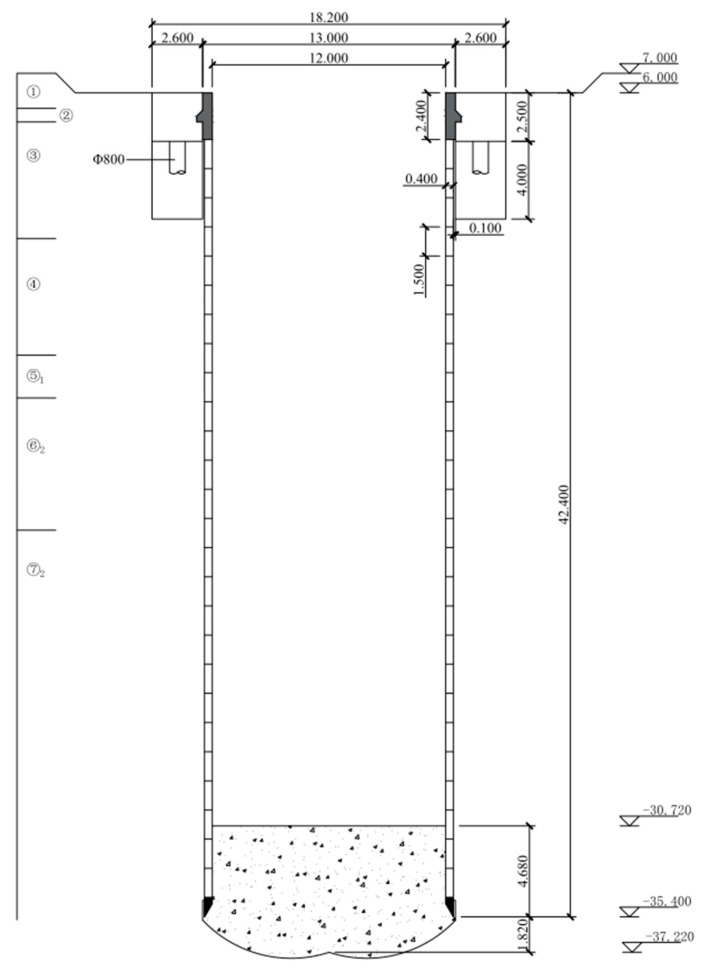
Shaft section layout (m).

In this project, the 17# shaft on connecting pipeline line is constructed by the VSM. Each ring of prefabricated caisson segments is made up of 6 identical segments, which are 1500 mm wide. It is connected by 12 radial and 18 longitudinal bolts. The segment concrete strength grade is C60 (the uniaxial compressive strength reaches 60 MPa of a national standard specimen after a curing duration of 28 d), and the impermeability grade is P12 (which can resist hydrostatic pressure of 1.2 MPa (equivalent to 120 m underwater) in a national standard specimen after a curing duration of 28 d). The design depth of the 17# shaft is 39 m, and the final sinking elevation of the blade foot is about −35.400 m. The shaft top is cemented with a 2.4 m height cast-in-place connection wall, which would be connected to the inside of the foundation ring beam after the shaft completely sinks in place. The foundation ring beam is used as the load-bearing structure of the VSM ground equipment during the shaft sinking process, as well as an anti-floating structure during the service stage. The section size of the foundation ring beam is about 2600 mm × 2500 mm with the inner and outer diameters of 13 m and 18.2 m, respectively. In total, 12 Φ800 mm diameter bored piles (cast-in-situ) are set under the foundation ring beam to bear the pressure load of the VSM equipment and the buoyancy of the shaft during the service stage. The section of the shaft structure is shown in Figure 2, technical parameters are shown in Table 1, and the formation parameters are shown in Table 2. 

## 4. Construction Process

**Figure 3:**
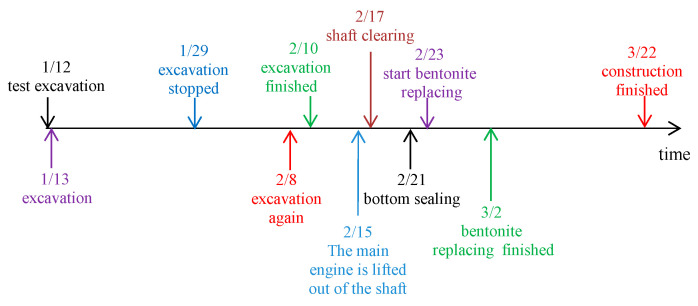
Construction node.

**Figure 4:**
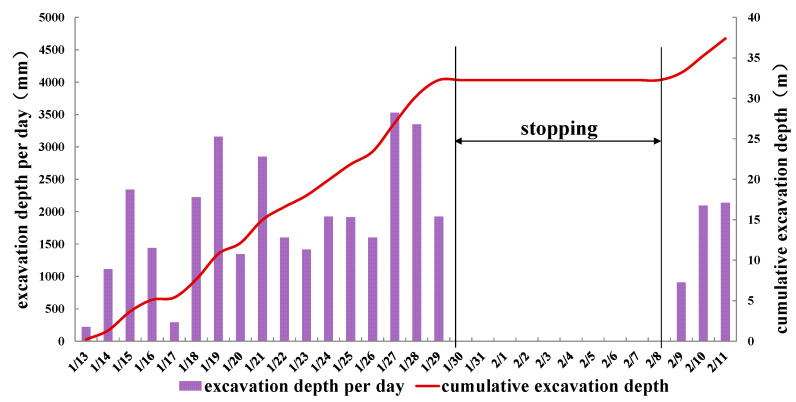
Construction progress.

The construction started at the end of 2021. After the preliminary work, such as equipment debugging and site leveling, the VSM officially initiated excavation on 13 January 2022. Due to the traditional Chinese Spring Festival holiday, the entire excavation process was divided into two stages. In total, 32.2 m was excavated for 17 days in the first stage from 13 January to 29 January, and 5.1 m was excavated for 3 days in the second stage from 8 February to 10 February. The excavation rate was 1.9 m per day on average, which was roughly the same throughout the excavation process. The bottom of the shaft was sealed on 21 February, the bentonite replacement was completed on 2 March, and the construction of the shaft bottom was completed on 22 March. The construction process is shown in Figure 3, and the construction excavation rate is shown in Figure 4.

## 5. Earth Pressure Design Theory in Shaft Lining

### 5.1. Theoretical Calculation of Earth Pressure

Lots of researchers discussed the earth pressure theories in shaft engineering in cohesionless soils and the saturated undrained soft clay [13,14]. They all agree that the active Rankine values at the greater depth are advisable in the design purpose, which is also consistent with the technical code for excavation engineering (DG/TJ 08-61-2018) [15] in Shanghai. In Prater’s [13] discussion, the earth pressure according to Berezantzev, reaches a limiting value asymptotically, which is much smaller than the Rankine value at greater depths, with a reduction in pressure at the greater depth, due to the arching action. Similar is known to exist for retaining walls not fulfilling the plasticity deformation conditions. While in the soft soil area, especially in Shanghai, as mentioned above, the large thickness (in this case, subsurface 20~40 m) soft mucky clay has poor permeability and high plasticity. Both with the plain assumptions of the Rankine earth pressure theory (the same as the Coulomb method for cohesionless soils), the active Rankine values are most conservative, and it would be advisable for design purposes. The Rankine earth pressure theory could be used to calculate the earth pressure in shaft design.

$$p_{ak}=\sigma_{ak}K_{ai}-2c\sqrt{K_{ai}}\;K_{ai}=tan^{2}\left(45{}^{\circ}-\varphi /2\right)\;\sigma_{o}=K_{0}\gamma h$$

Here, $p_{ak}$ is the active earth pressure on the outside soil of support structure (kPa); $\sigma_{ak}$ is the vertical stress in the soil layer at the calculated position outside the support structure (kPa); φ is the internal friction angle (°); $\sigma_{o}$ is the static earth pressure (kPa); *γ* is the weight of soil (kN/m^3^); *h* is the thickness of the soil (m).

### 5.2. Theoretical Calculation of Surface Deformation

Bowles [16] proposed a method to predict the ground surface settlement of the cohesive soil layer without considering the consolidation settlement. The calculation is as follows [17]:

(1) Calculate the lateral deformation of the support structure;
(2) Calculate the horizontal volume Vs of soil outside foundation pit;
(3) Estimate the influence area *D* of soil settlement outside foundation pit;
  Vs=(He+Hd)tan(45°−φ2)
  where $H_{e}$ is the excavation depth (m); $H_{d}$ is the excavation width or diameter (m).
(4) Calculate the maximum surface subsidence δ
  δ=(VSD)(xD)2
  where *x* is the distance from the calculation point to the support structure.

## 6. Shaft Monitoring

### 6.1. Monitoring Purpose 

In this project, on the one hand, monitoring work was implemented in order to ensure the construction quality and safety of shaft; the monitoring data were used to timely adjust the shaft excavation and the sinking speed to prevent shaft instability. On the other hand, the surrounding environment impact was also monitored by ground settlement and deep layered deformations at different locations. 

### 6.2. Monitoring Content

In this project, the deformation and stress conditions of the soil surrounding shaft were monitored. Total stations were used to monitor the ground settlement around the shaft; fixed inclinometers were used to monitor the horizontal displacement of stratums around the shaft along depth; and the buried earth pressure gauges were used to monitor the soil stress state.

**Figure 5:**
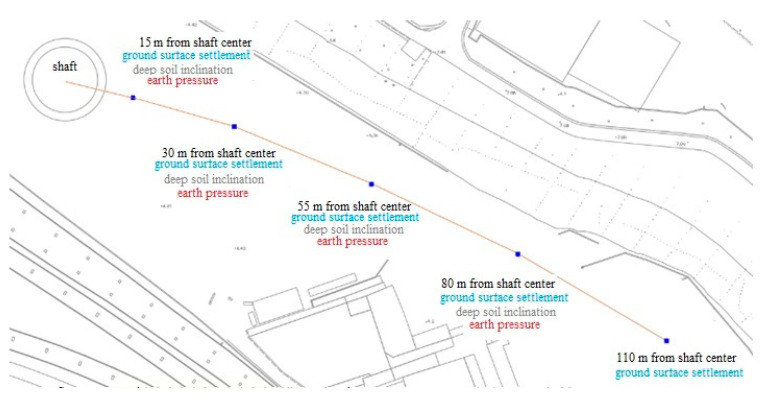
Layout of monitoring points.

In the construction site, 5 ground surface settlement monitoring points and 5 deep soil inclination observation points are arranged at 15 m, 30 m, 55 m, 80 m, and 110 m from the center of shaft, with depths of 5 m, 15 m, 25 m, 35 m, and 45 m; the positions of the observation points are shown in Figure 5. In total, 5 earth pressure gauges were buried at the depths of 5 m, 15 m, 25 m, 35 m, and 45 m, at the distance of 15 m from the shaft center.

### 6.3. Analysis of Measured Deformation during Shaft Construction

#### 6.3.1. Ground Settlement Analysis

**Figure 6:**
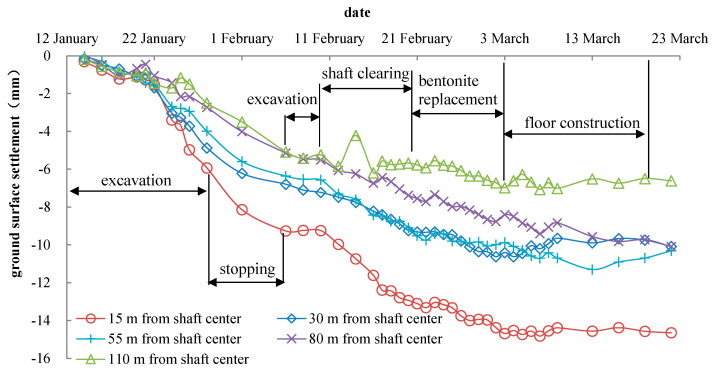
Monitoring results of ground settlement.

The ground settlement results of 15 m, 30 m, 55 m, 80 m, and 110 m from the shaft center are shown in Figure 6. The ground settlement was greater near the shaft. It was not obvious in the first 7 days of the excavation and increased rapidly after that. When the shaft excavation was suspended, the ground surface continued to settle for a period of time (7 days). This reveals that, in the soft clay layer with high plasticity and poor permeability, the ground settlement continued even without the disturbance of excavation. It will increase for a period of time after the excavation ends. This phenomenon is well-known to exist in the traditional slow excavation in the deep foundation pit of retaining walls (diaphragm walls), in which large deformation always happened if the support could be adjusted in time. Monitoring, as well as engineering control, during this period are still vital. After the bottom sealing was completed, the ground surface settlement increased slightly during the bentonite replacement stage; there was no significant change in the ground settlement during the shaft floor construction stage. 

It can be discovered from Figure 6 that the excavation of the VSM has a significant impact on the ground settlement within 30 m from the shaft center, which forms a settlement tank. By the end of the shaft construction, the maximum ground settlement was about 14.56 mm at 15 m away from the shaft center and the minimum ground settlement was about 6.62 mm at 110 m away from the shaft center. In total, the effect of the shaft construction on the ground settlement was relatively small under the VSM construction speed, especially in the shallow part, even including the additional settlement due to temporally stopping during the holidays. 

#### 6.3.2. Deep Layered Displacements Analysis

**Figure 7:**
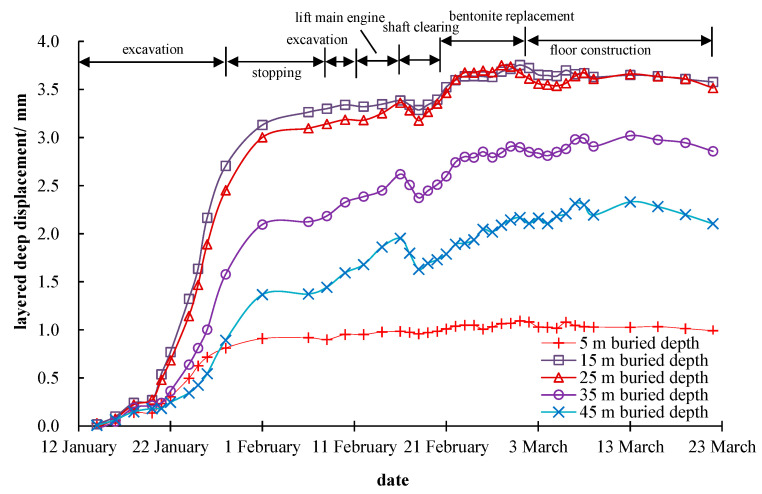
Monitoring results of the deep layered displacement at 15 m away from the shaft center.

It can be noticed from Figure 7 that the maximum deep layered displacement of the stratum was about 3.74 mm at 15 m away from the shaft center. During the construction of the shaft, the deep layered displacements increased continuously, and the growth rate was getting faster and faster. Except for the stratum 5 m deep underground, the deeper the depth, the smaller the horizontal displacement. (The small horizontal displacement of the stratum 5 m depth underground may be due to the surface hardening around the shaft and the construction equipment placed on the ground surface, which constrained the horizontal deformation near the ground surface to a certain extent.)

**Figure 8:**
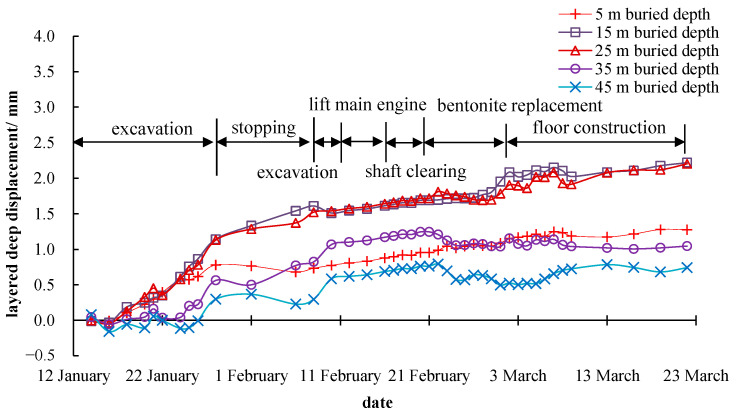
Monitoring results of the deep layered displacement at 30 m away from the shaft center.

**Figure 9:**
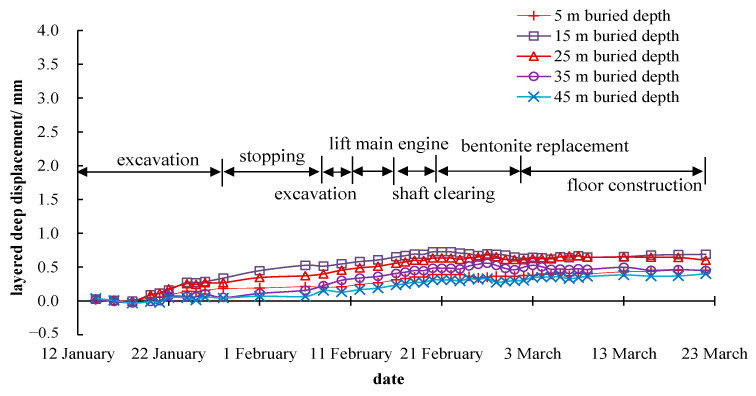
Monitoring results of the deep layered displacement at 85 m away from the shaft center.

When the excavation ends, the deep layered displacement of the monitoring point at 15 m away from the shaft center immediately stopped increasing. It can be inferred that the horizontal displacement has a strong time correlation with the excavation operation. From Figure 8 and Figure 9, the maximum horizontal displacements of the stratum, measured by the inclinometer pipes 30 m and 85 m away from the shaft center, were about 2.20 mm and 0.63 mm. The horizontal displacement started to increase after 3–5 days after the construction began and did not stop rising when the excavation was completed. The horizontal displacement growth rate was roughly the same, and there was almost no accelerated growth trend. The reason for this phenomenon is that the deep viscoplastic soft soil layer in the construction site prolonged the deformation loading transfer time and weakened the deformation strength. Comparing the measurement results of the horizontal displacement at the same depth and different distances from the shaft, it could be figured out that the closer the distance to the shaft, the greater the horizontal displacement caused by the excavation. The maximum horizontal displacement is about 4.71 mm at 15 m from the shaft center and around 15 m in depth. In general, the displacement and deformation during the whole construction process were small, which meets the requirements of the technical code for excavation engineering (DG/TJ 08-61-2018) [15] for Shanghai.

#### 6.3.3. Lateral Earth Pressure Monitoring and Analysis

**Figure 10:**
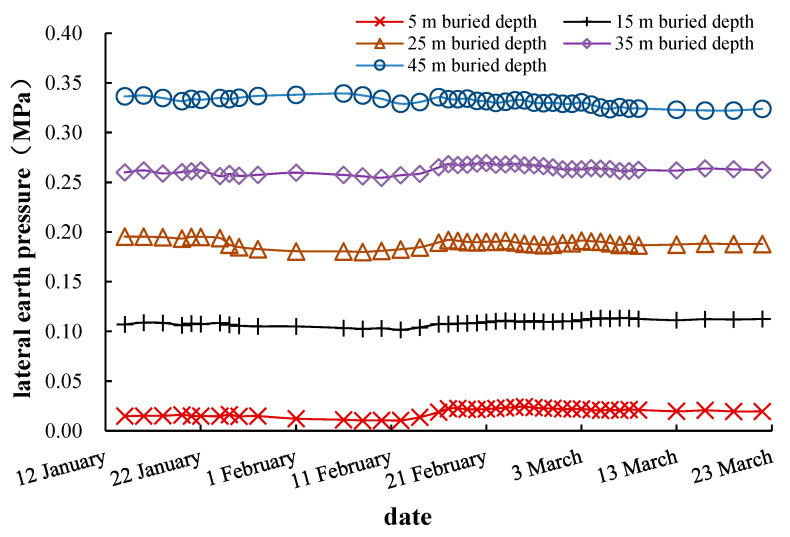
Monitoring results of lateral earth pressure at 15 m away from the shaft.

The lateral earth pressure monitoring results are shown in Figure 10. It can be figured out from Figure 10 that the lateral earth pressure decreased slightly during the excavation stage, about 5%. The overall lateral earth pressure changes little during the whole construction process, indicating that the shaft construction had a small disturbance on the stress state of the surrounding soil.

## 7. Numerical Simulation and Analysis 

**Figure 11:**
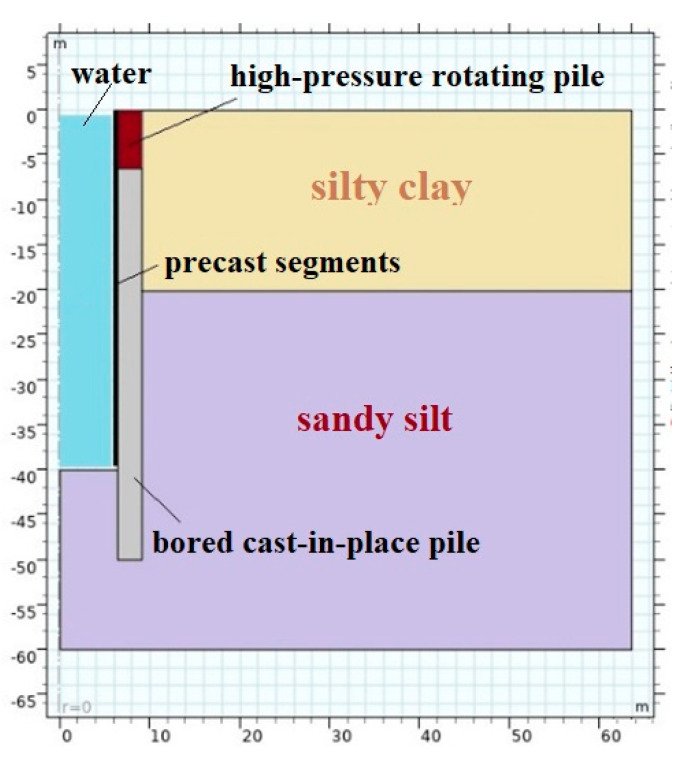
Numerical simulation model.

An axisymmetric steady state numerical simulation model is established to analyze the construction process of the VSM. Due to the fast construction speed and mostly poor permeability of Shanghai soft soils, the undrained condition is considered, and the consolidation of soil is neglected during the VSM excavation. However, before the excavation, a Biot consolidation model was used for the initial stress state computation under self-weight. This process is called self-weight balance, in which it calculates and equilibrates the initial ground stress and makes the soil model fully consolidated, and the deformation gets stable under the self-weight, to form the initial stress field for the subsequent VSM construction model. In VSM excavation, fluid-solid coupling is considered, in which an elastic-plastic Mohr-Coulomb model governs stress-strain field; and Darcy’s law governs the seepage field. It is carried out by effective stress. The change of water head by the seepage in the shaft causes the change of the pore water pressure in the soil layers and then changes the effective stress and causes deformation. The soil layers in the construction site are: ① plain fill/flush fill, ② sandy silt, ③ clay, ④ silty clay, ⑤_1_ clay, ⑥_2_ clay mixed with silt, and ⑦_2_ sandy silt. Considering that the engineering properties of the ② sandy silt, ④ silty clay and ⑤_1_ clay layer are roughly the same, and the effects of the thinner ① plain fill/flush fill and ② sandy silt are ignored; this model generalizes the stratum as a combination of ④ silty clay and ⑦_2_ sandy silt. The simulation model is shown as Figure 11, soil parameters are shown in Table 3, and structure parameters are shown in Table 4.

The consolidation of soil was completed before excavation in the numerical simulation model. Resultantly, the whole VSM model has an initial stress and strain field under self-weight. Furthermore, the shaft excavation and segment sinking are calculated at the same time every 5 m. As explained above, consolidation is not considered during the excavation process because of the rapid construction speed, short construction time, and low permeability of soils. The normal displacement of the side boundary and the lower boundary of the model are constrained. The hydraulic pressure is applied to the permeable excavation face and the inner wall of the prefabricated concrete segment. On the ground surface, the gravity of the prefabricated segment in the shaft is loaded on the concrete ring beam, and the pavement load of 5 kPa is loaded within 50 m from the outer wall of the shaft. 

### 7.1. Simulation Results of Ground Settlement

**Figure 12:**
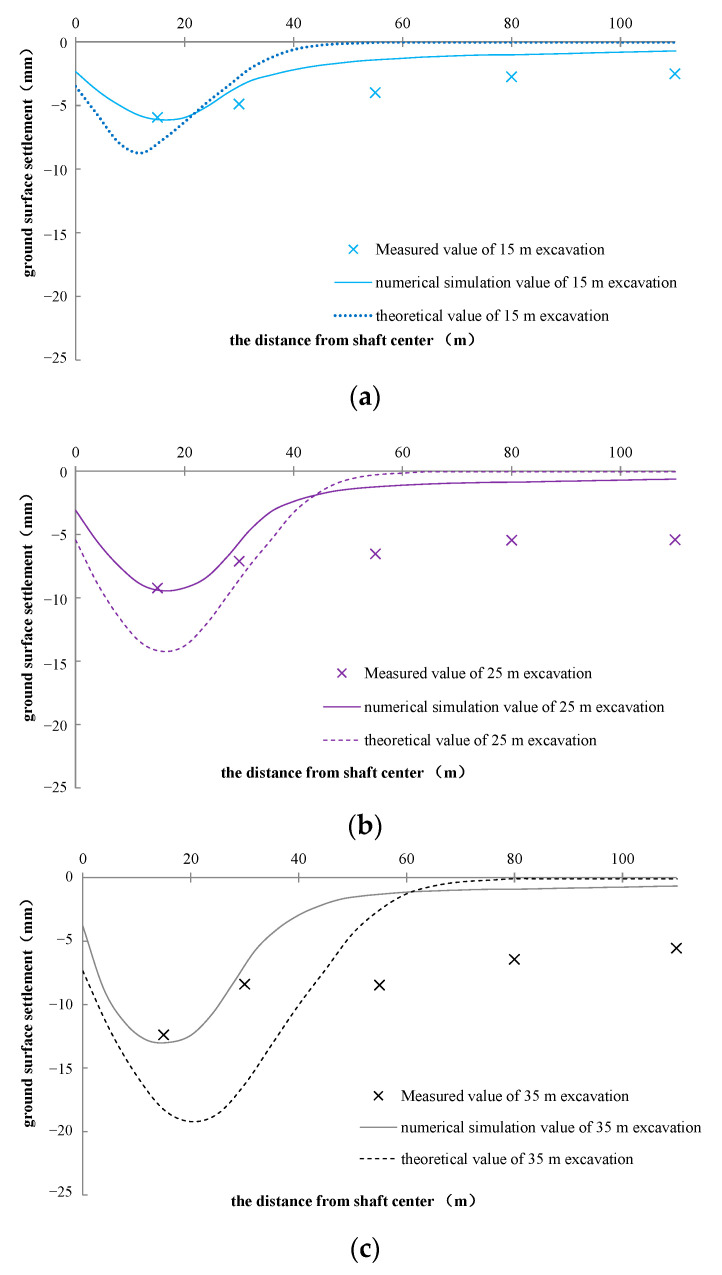
Simulation results of surface settlement ((**a**), 15 m excavation; (**b**), 25 m excavation; (**c**), 35 m excavation).

Ground settlements of simulation results during 15 m, 25 m, and 35 m excavation, along different distances from the shaft center, are presented in Figure 12, combined together with field measurements and theoretical calculations. They all show that the ground settlement is mainly concentrated in the range of 30 m from the shaft center. The ground settlements all behave as a shape of a spoon type. As the excavation increases, the maximum ground settlement gets slightly larger, but the location is almost the same, all-around 15–20 m distance from the shaft center. The numerical simulation results in the range of 30 m from the shaft center are roughly the same as the field measurements. When the excavation of the shaft was basically completed (35 m excavation depth), the ground surface settlement 15 m from the shaft center obtained by numerical simulation is about 13.6 mm, and the measured value is about 13.4 mm. The simulation value is relatively consistent with the measured value; on another aspect, the simulation results also demonstrate the validity and effectiveness of the field measurement points design. The theoretical calculation result is about 19.7 mm, which is larger than the simulation and measured values. These results may be due to the small diameter of the VSM shaft, and the annular lining structure has great bending resistance in the radial direction. However, the stress and deformation theory (explained above) simplified segments into a two-dimensional structure (radial and axial directions), which reduces the radial bending resistance, thus making the theoretical calculation settlement larger. The field measurement shows that there is still a lot of ground settlement beyond 30 m away from shaft center, which is obviously larger than the numerical simulation and theoretical calculation results. The reason for this phenomenon may be that the construction site has a lot of construction facilities and building materials stacking in this range. These temporary overloadings greatly increase the ground settlement at this construction site. The numerical simulation and theoretical calculation are insufficient to estimate the construction overload, resulting in the phenomenon that the measured value is obviously larger than the simulated and calculated values. Both the simulation and measurement results show that the ground settlement caused by the VSM excavation is not greater than 15 mm, which is relatively small compared with other underground structure construction, inferring that the VSM construction has little impact on the ground settlement.

### 7.2. Analysis of Uplift Deformation on Shaft Excavation Face 

**Figure 13:**
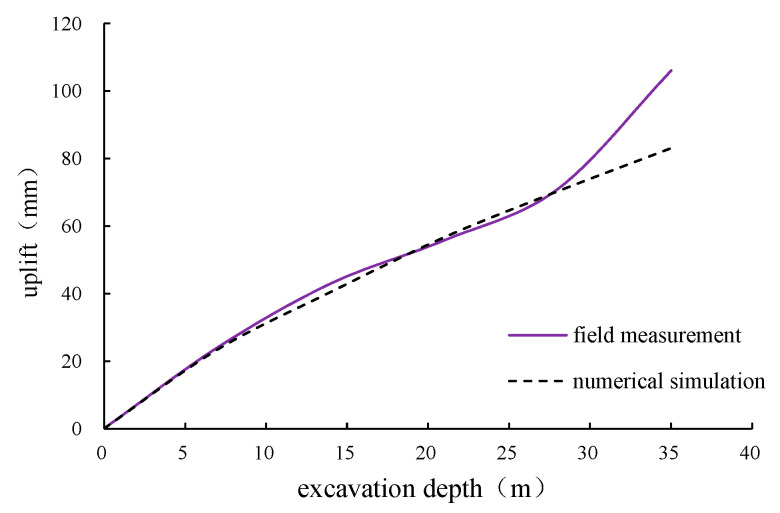
Uplift simulation results near the excavation face.

The vertical uplift results of the soil near the excavation surface by simulation are shown as Figure 13, compared with field measurements. Both the numerical simulation and field measurement show that, during the excavation, a certain uplift would occur near the excavation surface; the deeper the excavation depth, the more obvious the uplift. The numerical simulation results of the vertical deformation near the excavation face are generally slightly larger than the field measurement results. The numerical simulation results of the excavation face uplift after shaft excavation are about 10.7 cm, while the measured result is about 8.6 cm. The reason for this phenomenon is that the caisson bottom soil has a certain elastoplasticity, causing a slow rebound rate. Under the rapid construction rate of the VSM, the foundation soil is not fully rebounded until the construction of the bottom sealing is completed. In general, the numerical simulation model is able to simulate the real excavation face uplift, and the results tend to be a good reference before the construction.

### 7.3. Analysis of Horizontal Displacement along Depth

**Figure 14:**
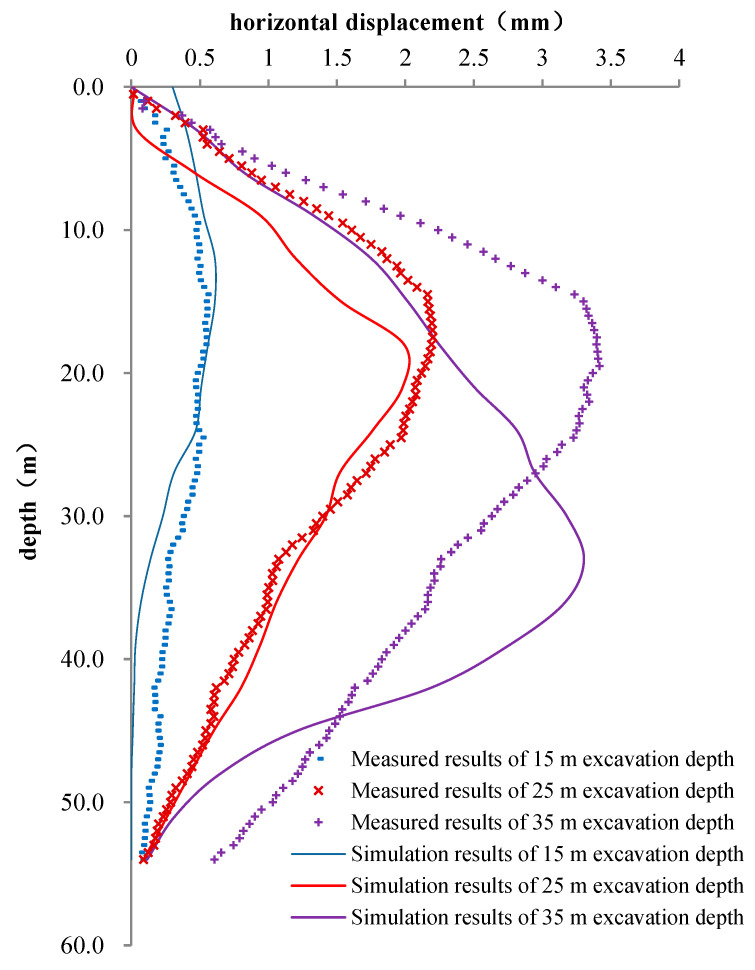
Simulation and measured results of the horizontal displacement of the shaft along depth of 15 m away from shaft center.

In the construction site, the horizontal displacement simulation and the measured results along the depth of 15 m away from the shaft center are shown in Figure 14. Comparing the numerical simulation with the field measurements, the simulation values of the horizontal displacement under different excavation depths are slightly smaller than the measurement values. When the construction is finished (35 m excavation), at 15 m away from shaft center, the simulation result for the maximum horizontal displacement is about 3.26 mm, while the field measurement value is about 3.45 mm, and the error is only 6%. However, the main difference here is that the numerical simulation shows that the maximum horizontal displacement is located at the depth of 30 m to 40 m, while the measured results show that the maximum deformation occurs at the depth of 15 m to 20 m. This is mainly because this depth of 15–20 m is at the interface of the soft clay and silt. When excavating in the upper soft clay layer, due to the thixotropy of the soft clay, it is necessary to grout the bentonite between the prefabricated segments and the soil to ensure the stability of the shaft. The parameters of bentonite need to be strictly controlled in this process. The soil parameter changes abruptly at the interface of the stratum, and the bentonite parameter fails to adjust in time, leading to a large deformation. This process is not simulated by the model, leading to differences in results.

### 7.4. Analysis of Lateral Earth Pressure

**Figure 15:**
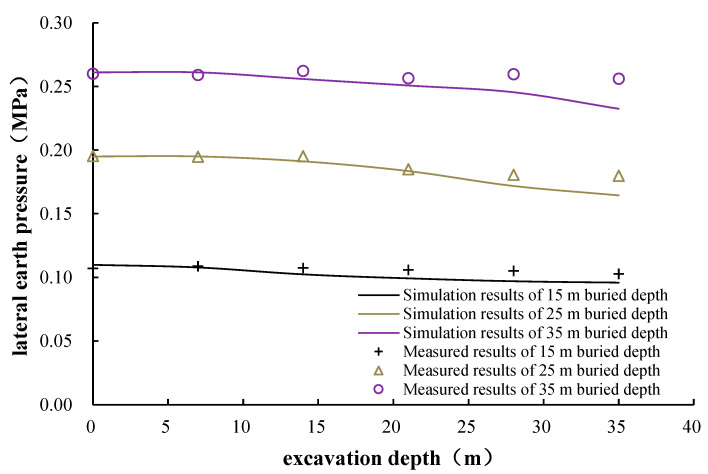
Simulation and measured results of lateral earth pressure at 15 m away from shaft center.

The lateral earth pressure results along the excavation depth at 15 m away from the shaft center by simulation are shown in Figure 15, compared with the field measurements. The numerical simulation results show that the lateral earth pressure decreases slightly during the excavation process; the lateral earth pressure at the construction ending is about 7% lower than that at the construction beginning. In general, there is little difference between the simulated and measured values of the lateral earth pressure along the excavation depth, and the numerical simulation could roughly simulate the earth pressure during the excavation process.

**Figure 16:**
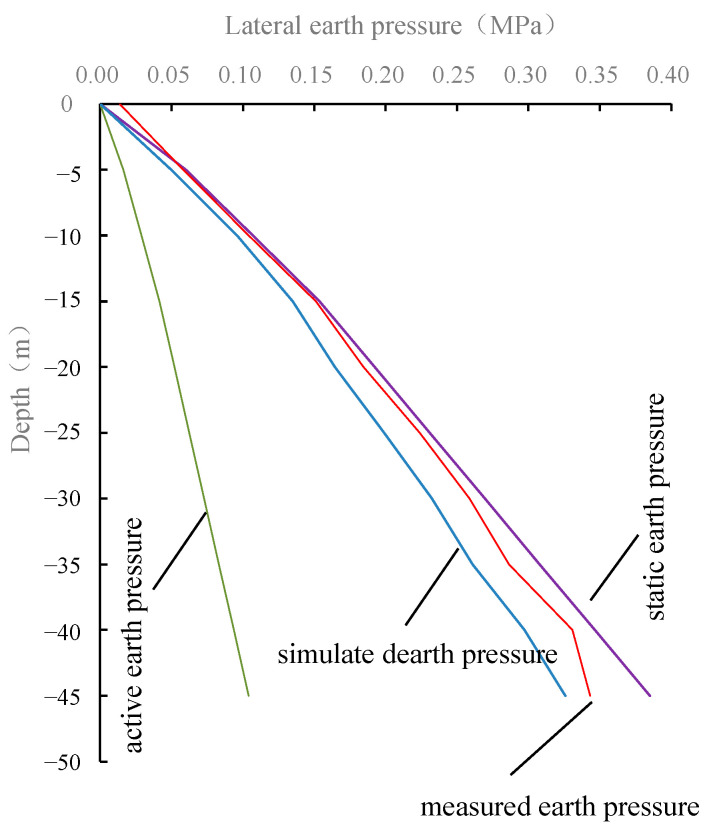
Simulation and measured results of lateral earth pressure at 15 m away from shaft center.

When the excavation finished, the numerical simulation, field measurement, and theoretical calculation of the lateral earth pressure at different depths of 15 m away from shaft center are shown in Figure 16. Both the numerical simulation results and the measured results show that the lateral earth pressure is slightly smaller than the static earth pressure and much larger than the active earth pressure. This shows that the horizontal deformation of shaft segments is relatively small during the excavation process, and the caisson soil is far away from the stress state that actively and completely squeezes the segments. The shaft excavation has little effect on the lateral earth pressure of the soil.

### 7.5. Stability Analysis during Shaft Excavation

During the traditional excavation of the shaft, the soil is probably at risk of instability [18]. The monolithic stability safety factor is an important evaluation index for the stability of the shaft excavation and the inrush risk on the excavation surface.

$$F_{s}=\frac{\sum \;R_{n}}{\sum \;T_{n}}$$

where $F_{s}$ is the stability safety factor; $R_{n}$ is the normal shear stress at a point on the sliding surface (kN); $T_{n}$ is the normal shear strength at a certain point on the sliding surface (kN).

**Figure 17:**
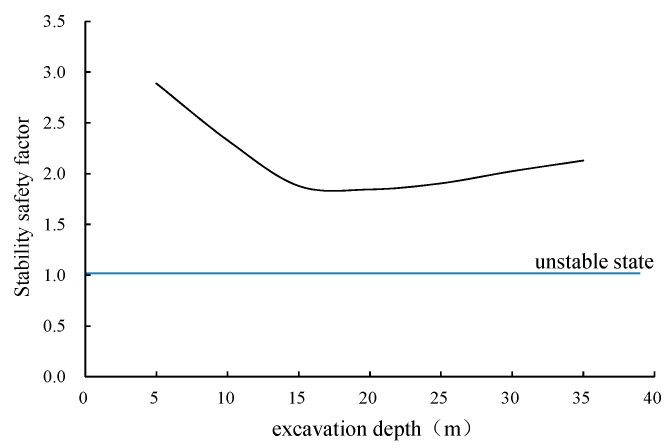
Stability safety factor of shaft excavation.

The numerical simulation model can automatically search for the most likely failure surface and calculate the stability safety factor of the shaft during the excavation process. The simulation results of the stability safety factor are shown in Figure 17. It can be seen from Figure 17 that during the excavation of the VSM shaft, the stability safety factors are all greater than 1.8, the overall risk of soil instability and water inrush is very small, and the construction of the VSM shaft is relatively safe due to the injection of water always during the construction. In the range of the excavation depth less than 15 m, the stability safety factor decreases continuously with the excavation of the shaft. The stability of the shaft is the worst when the excavation depth is in the range of 15–20 m. When the excavation depth exceeds 20 m, the stability increases slightly. The reason for this phenomenon is that, with the increase in the excavation depth, the sliding force provided by the overlying soil is far greater than the anti-sliding force provided by it. The stability of the structure is in the most dangerous state when the excavation reaches the interface between the soft clay and silt. However, the scope of the most dangerous failure surface expands when the depth exceeds a certain depth, which inhibits the tendency of the soil to become unstable. Then, the deeper the excavation depth, the smaller the hydraulic gradient of the pore water in the soil infiltrating into the shaft, and lower the seepage force, which also increases the stability in the deeper stage of the excavation. In general, the risk of soil instability and water inrush during the excavation process of the shaft is very small. The VSM is a relatively reliable construction method for the shaft when full of water inside the caisson. The only attention that should be paid is when the water discharged; but at that moment the caisson bottom has already been sealed. 

## 8. Discussion

The soft clay is widely distributed in the Shanghai area. Because soft clay has a strong viscoplasticity and also poor permeability, the construction speed of the underground space structure would greatly affect the soil deformation. The longer the construction time, the greater the soil deformation. The construction speed of in-situ assembling caisson technology is significantly faster than that of the cast-in-place concrete structure, which reduces the disturbance of the construction on the surrounding soil.

The process of the underground space excavation inevitably encounters temporary stopping. Engineering practice shows that, in the Shanghai area where soft clay is widely distributed, the construction stopping of underground buildings would still bring certain deformation and settlement to the environment because of creep [19,20]. The VSM is no exception. However, because the VSM construction is an undrained operation, the stress disturbance to the surrounding soil is very small, and the safety and stability of the shaft during the stopping period are relatively high. The construction of the entire VSM is preferred to the continuous operation to minimize the creep deformation. During the temporary stopping, the water level in the shaft should be raised to reduce settlement. The relevant parameters of bentonite grouting should be adjusted in time to reduce the lateral deformation of the soil layer when excavating to the interface of stratum, which ensures the construction safety and controls the deformation.

## 9. Conclusions

This paper takes the Shanghai Zhuyuan Bailonggang Sewage Connecting Pipe Project as the engineering background and briefly describes the technical aspects of in-situ assembling caisson technology and construction procedures of VSM. Ground settlements and deep layered displacements were monitored during the VSM construction, and corresponding theoretical calculations and numerical simulations were compared. Several important conclusions are drawn as follows:

(1) The VSM has the advantages of deep excavation depth, fast construction speed, small site usage, high economic efficiency, and strong stratum adaptability. It is an excellent construction method for urban vertical excavation using prefabricated in-situ assembly to build caisson, also with surrounding environmentally-friendly merit. The ground settlement and deep layered deformation during construction, both from measured results and numerical simulation, show that the VSM could be constructed underwater with little effect on the surrounding environment and high construction safety. It has obvious advantages in soft soil areas, which generally require engineering dewatering.

(2) Ground settlement is mainly concentrated within 30 m away from the shaft center. The maximum ground settlement caused by VSM excavation is about 15.2 mm and the maximum horizontal displacement is about 3.74 mm during the excavation depth of 40 m. When the shaft is excavated to the soil interface, the horizontal displacement of the stratum is the largest. The effect of shaft excavation on the lateral earth pressure is very small. The lateral earth pressure is slightly reduced by about 7% during the construction process. Compared with other construction techniques, its disturbance to the surrounding soil is really small.

(3) During the VSM construction excavation of shaft, the stability safety factors are all greater than 1.8. The risk of soil instability and water inrush during the shaft construction excavation is very small. The VSM is a relatively safe and reliable shaft construction method underwater, without the requirement of dewater in the soft soil area. The seepage stability of the VSM should be paid attention when water discharging.

(4) The numerical simulation results of the ground settlement, deep layered displacements, the uplift of the excavation bottom, and the lateral earth pressure are relatively accurate, verified by field measurement. It can effectively simulate the construction site conditions. The traditional foundation pit stress and deformation theory is relatively accurate for the calculation of the earth pressure, but the calculation results of the ground deformation have larger errors to field measurements, so the estimation accuracy is not high.

(5) The shaft should be constructed continuously to avoid the creep deformation of the soft clay during temporary stopping. If one has to, the water level in the shaft should be raised. The relevant parameters of bentonite grouting should be elaborately controlled in time to reduce the lateral deformation of the soil layer when excavating to the interface of stratum for stability control. 

## Figures and Tables

**Table 1:** Technical parameters in VSM construction.

Shaft Structural Parameters	Mechanical Arm Parameters	Excavation and Assembly Speed	Bentonite Parameters
Excavation depth: 43.22 m Excavation radius: 6.5 m Inside diameter of shaft: 6 m Over excavation depth: 15~20 cm Prefabricated segments: C60 concrete Maximum depth: 120 m	Elongation: 0~1000 mm Swing scope: −10°~+47° Rotation scope: ±190°	Up to 4.5 m/d	Marsh funnel viscosity: 90 Ms/L Static yield point: 40 N/mm² API filtration: up to 20 mL

**Table 2:** Soil layer characteristics.

Number	Soil Layer	Unit Weight/kg/m^3^	Void Ratio	Compression Modulus /MPa	Cohesion /kPa	Internal Friction Angle/°	Thickness/m
①	plain fill/flush fill	1800	1 *	2 *	10 *	10 *	1.8
②	sandy silt	1840	0.85	5.5	5	30.5	0.7
③	clay	1780	1.11	2.2	12	17.5	6.0
④	silty clay	1690	1.38	2.1	12	12	6.0
⑤_1_	clay	1800	0.97	4.5	18	17	2.2
⑥_2_	clay mixed with silt	1820	0.9	6	19	19	6.8
⑦_2_	sandy silt	1890	0.76	13.5	3	34	35.5

Note: * is empirical value.

**Table 3:** Soil parameters in numerical simulation model.

Soil Layer	Unit Weight /kg/m^3^	Void Ratio	Young’s Modulus/Mpa	Cohesion/kPa	Internal Friction Angle/°	Permeability /cm/s
silty clay	1690	1.38	6.57	12	12	1.43 × 10^−7^
sandy silt	1890	0.76	19.2	3	34	4.69 × 10^−5^

**Table 4:** Structure parameters in numerical simulation model.

Material	Unit Weight/kg/m^3^	Young’s Modulus/Mpa	Poisson’s Ratio
precast segments	2300	2.5 × 10^4^	0.2
high-pressure rotating pile	1800	20	0.2
bored cast-in-place pile	2300	3.15 × 10^4^	0.2

## Data Availability

Not applicable.
